# Editorial of Special Issue “Microalgal Molecules and Enzymes”

**DOI:** 10.3390/ijms222413450

**Published:** 2021-12-15

**Authors:** Chiara Lauritano, Assunta Saide

**Affiliations:** Ecosustainable Marine Biotechnology Department, Stazione Zoologica Anton Dohrn, Villa Comunale, 80121 Napoli, Italy; assunta.saide@szn.it

Microalgae are photosynthetic unicellular organisms that can be found in very different environments, both terrestrial and marine, including extreme environments such as cold, hot and high/low salinity [1]. This capability results in a huge diversity of species, as well as the production of compounds which are of great biotechnological interest. Some species can be found both in marine and fresh water habitats, such as the model species *Chlamydomonas* [2]. Microalgae have attracted attention from researchers worldwide because they have been shown to be a valuable source of bioactive molecules and enzymes, with applications beneficial for human health and the environment (Figure 1). Microalgae can be cultivated on large scales under controlled eco-friendly conditions in enclosed photobioreactors (e.g., tubular, flat plate, twin-layers, inclined tubular, helical and column bioreactors) with relatively small quantities of micro- and macro-nutrients. Microalgae have been shown to be excellent sources of several bioactive molecules such as lipids, carbohydrates, vitamins, pigments, and a series of other compounds. In addition to pure molecules, microalgal raw extracts and fractions have been found to exhibit various biological activities, such as anticancer, anti-microbial, anti-epilepsy, anti-inflammatory, and immunomodulatory activities.

In this Special Issue, Saide et al. [1] provides an overview on chemical structures, biological activities, the known mechanisms of action, and enzymatic pathways involved in the synthesis of marine microalgal metabolites. They reviewed the different classes of microalgal-derived compounds which have been identified, such as pigments (e.g., fucoxanthin, β-carotene, astaxanthin, violaxanthin, lutein, and minor carotenoids), polyphenols, polysaccharides, lipids, glycolipids, steroids, oxylipins, proteins/peptides, and polyketides/macrolides (including microalgal toxins). Several microalgal molecules, as well as raw extracts and fractions, have been shown to exert specific biological activities, such as anticancer [3], anti-inflammatory [4], immunomodulatory [5] anti-diabetes [6], antioxidant [7], anti-tuberculosis [8], anti-epilepsy [9], anti-hypertensive [10], anti-atherosclerosis [10], and anti-osteoporosis [10] activities.

Another review reported in this Special Issue is focused on the carotenoid fucoxanthin derived from brown seaweeds and microalgae [11] and, in particular, focuses on the anticancer properties. The authors reviewed that fucoxanthin is able to arrest the cell growth of various cancer cells, induce apoptosis, and/or autophagy both in cells and in animal models of cancer (e.g., lung, liver, duodenal, colorectal, breast, cervix, lymphoma, melanoma, sarcoma, and glioblastoma cancer models). It is also able to inhibit metastasis-related migration, invasion, epithelial–mesenchymal transition, and angiogenesis, and it was suggested that, in combination with other drugs usually used in anticancer treatments, it may be useful for reducing drug resistance [11].

Drug discovery from microorganisms, especially those which are easily cultivable, has attracted considerable attention in recent years; it has been demonstrated that different culturing conditions and/or stress exposure, including variations in temperature, light, nutrients, and incubation with predators, may influence microalgal bioactivities and the production of natural compounds [12,13,14]. This approach is known as the “OSMAC” approach (one strain, many compounds) triggering the production of microalgal molecules with potential benefits for human health. In a recent paper of Su and co-workers [15], presented in this Special Issue, authors focused on the oleaginous microalgae species *Chlorococcum sphacosum* GD, a promising feedstock for biodiesel production from soil. They determined biomass and lipid algal content under different NaCl concentrations and studied the metabolic mechanism of lipid production of *C. sphacosum* GD under salt stress by using the transcriptome sequencing approach. They showed that when the salt concentration increased in culture medium, the lipid content increased but the algal biomass decreased. In addition, between the differentially expressed genes (of which 2051 were up-regulated and 1835 were down-regulated genes), genes related to fatty acid biosynthesis (e.g., acetyl-CoA carboxylase, β-ketoacyl-ACP reductase, and β-Hydroxyacyl-ACP dehydratase catalysis) were significantly up-regulated, whereas some genes related to fatty acid degradation were down-regulated. The overall results showed that salt stress may alter lipid accumulation in *C. sphacosum* GD with interesting possible biodiesel applications.

With the advent of omics technologies and gene-editing, several genomes and transcriptomes have been sequenced, and genetic manipulation techniques have been applied, opening new scenarios for the production of valuable products with applications in various sectors, such as nutraceutical and pharmaceutical sectors, but also biodiesel production and bioremediation [16]. Kim and co-workers [17], in this Special Issue, report very recent results on the editing of the genome of the microalga *C. vulgaris* UTEX395 using clustered, regularly interspaced, short palindromic repeats-associated protein 9 (CRISPR-Cas9) system targeting nitrate reductase and adenine phosphoribosyltransferase. The success of using this approach was demonstrated by expression levels of the correspondent proteins and through growth analysis under specific nutrient conditions, by avoiding the use of exogenous selection markers (e.g., antibiotic resistance genes) and proposing the development of new transgenic lines. *Chlorella* is a microalgal species used in food and feed sectors and, together with other few species, such as *Dunaliella*, has received the safe (GRAS) status, which implies that, according to the U.S. Food and Drug Administration (FDA), they are “safe to consume” [1]. In addition, it is considered as a feasible cell factory for molecules of interest.

As editors of this Special Issue, we hope that it will provide a valuable reference source for all researchers interested in microalgal molecules and enzymes.

## Figures and Tables

**Figure 1:**
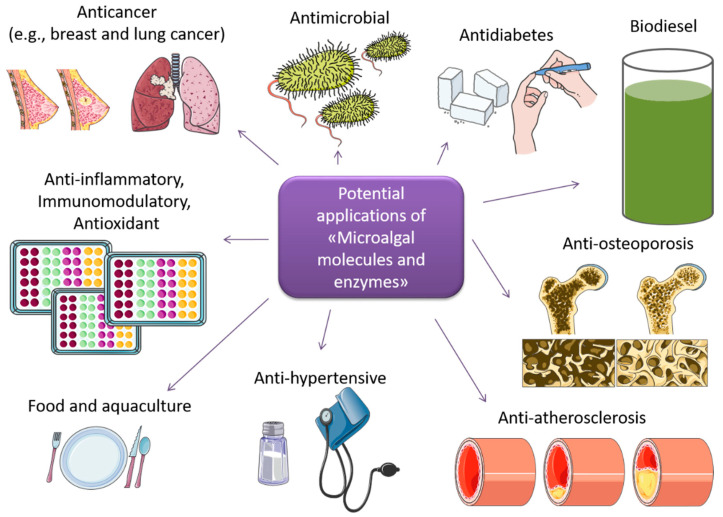
Possible applications of microalgal molecules and enzymes.

## Data Availability

Not applicable.
